# Evaluation of Matrix Metalloproteinases, Cytokines and Their Potential Role in the Development of Ovarian Cancer

**DOI:** 10.1371/journal.pone.0167149

**Published:** 2016-11-30

**Authors:** Mahmood Rasool, Arif Malik, Muhammad Abdul Basit Ashraf, Gulshan Parveen, Shazia Iqbal, Irfan Ali, Mahmood Husain Qazi, Muhammad Asif, Kashif Kamran, Asim Iqbal, Saima Iram, Sami Ullah Khan, Mohammad Zahid Mustafa, Ahmad Zaheer, Rozeena Shaikh, Hani Choudhry, Mohammad Sarwar Jamal

**Affiliations:** 1 Center of Excellence in Genomic Medicine Research (CEGMR), King Abdulaziz University, Jeddah, Saudi Arabia; 2 Institute of Molecular Biology and Biotechnology (IMBB), The University of Lahore, Lahore, Pakistan; 3 Akhuwat-Faisalabad Institute of Research Science and Technology, Faisalabad, Pakistan; 4 Centre for Research in Molecular Medicine (CRIMM), The University of Lahore, Lahore, Pakistan; 5 Department of Biotechnology, BUITEMS, Quetta, Pakistan; 6 Faculty of Life Sciences, University of Balochistan, Quetta, Pakistan; 7 Bolan Medical Hospital, Quetta, Pakistan; 8 Department of Botany, Women University of Azad Jammu & Kashmir, Bagh, Pakistan; 9 CASVAB, University of Balochistan, Quetta, Pakistan; 10 National Institute for Biotechnology & Genetic Engineering, Faisalabad, Pakistan; 11 Department of Biochemistry, Faculty of Science, Center of Innovation in Personalized Medicine, King Fahd Center for Medical Research, King Abdulaziz University, Jeddah, Saudi Arabia; 12 King Fahd Medical Research Center, King Abdulaziz University, Jeddah, Saudi Arabia; University of South Alabama Mitchell Cancer Institute, UNITED STATES

## Abstract

**Background:**

Ovarian cancer is the 5^th^ most common cause of deaths in the women among gynecological tumors. There are many growing evidences that stress and other behavioral factors may affect cancer progression and patient survival. The purpose of this study is to determine the key role of matrix metalloproteinases (MMPs), and cytokines in the aggregation and progression of ovarian cancer.

**Methodology:**

Stress variables (MDA, AGEs, AOPPs, NO), profile of antioxidants (SOD, Catalase, Vitamin E & A, GSH, GRx, GPx) and inflammatory biomarkers (MMP-9, MMP-2, MMP-11, IL-1α and TNF-α) were biochemically assessed from venous blood of fifty ovarian cancer patients and twenty healthy control subjects. The results of all parameters were analyzed statistically by independent sample t-test.

**Results:**

The results of the study demonstrated that the levels of stress variables like MDA (3.38±1.12nmol/ml), AGEs (2.72±0.22 ng/ml), AOPPs (128.48±27.23 ng/ml) and NO (58.71±8.67 ng/ml) were increased in the patients of ovarian cancer as compared to control individuals whereas the profile of antioxidants like SOD, Catalase, Vitamin E, Vitamin A, GSH and GRx were decreased in ovarian cancer patients (0.11±0.08 μg/ml, 2.41±1.01μmol/mol of protein, 0.22±0.04 μg/ml, 45.84±9.07μg/ml, 4.88±1.18μg/ml, 5.33±1.26 μmol/ml respectively). But the level of GPx antioxidant was increased in ovarian cancer patients (6.58±0.21μmol/ml). Moreover the levels of MMP-9 (64.87±5.35 ng/ml), MMP-2 (75.87±18.82 ng/ml) and MMP-11 (63.58±8.48 ng/ml) were elevated in the patients. Similarly, the levels of various cytokines TNF-α and IL-1α were also increased in the patients of ovarian cancer (32.17±3.52 pg/ml and 7.04±0.85 pg/ml respectively).

**Conclusion:**

MMPs are commonly expressed in ovarian cancer which are potential extrapolative biomarkers and have a major role in metastasis. Due to oxidative stress, different cytokines are released by tumor associated macrophages (TAMs) that result in the cancer progression. Consequently, tissue inhibitors of matrix metalloproteinases (TIMPs) are the valuable therapeutic approaches to complement conservative anticancer strategies.

## Introduction

Ovarian cancer is one of the foremost causes of death among gynecological tumors [1–3]. It has been frequently called as “silent killer” because of non-specific symptoms and usually diagnosed at later stages because of non-reliable screening for early detection [4]. In worldwide, approximately 239,000 cases of ovarian cancer were estimated in 2012, with incidence rates different all across world. Pakistan has one of the highest rates of ovarian cancer, though accurate prevalence of ovarian cancer is not known, but it is fourth frequent cancers among women [5]. The precise reason of ovarian cancer in Pakistan is not known but it has been considered that it is due to the germ line mutations in BRCA1 and BRCA2 genes [6]. Currently, no method has been established for early detection, thus variable risk factors (smoking, hormonal replacement therapy, obesity, ionizing radiation, occupational hazards etc.) should be controlled to achieve primary prevention to lessen the ovarian cancer burden.

Reactive oxygen species (ROS) mediated oxidative insult is mainly taken as the disturbance between free radicals generation and competency of antioxidant defense system.

Matrix metalloproteinases (MMPs) are the potent mediators for cancer development and metastases that may be associated with gynecological cancer survival [7, 8]. Normally MMPs are produced in very low amount and are implicated in tissue remolding processes like ossification, embryonic development, placental development, and wound healing. While in disease conditions, the defective regulation leads to the elevated levels and involves in different cancers and rheumatoid arthritis etc. Its activation can be induced by inflammatory growth factor and cytokines particularly in most malignant cells. Whereas it can be inactivated by different types of inhibitors such as tissue inhibitors of metalloproteinases (TIMPs), α-2-macroglobulin and synthetic matrix metalloproteinase inhibitors (MMPIs)[8].

Cytokines and chemokines act as autocrine and paracrine growth factors in promoting malignant progression and their expression is raised in response to infection or injury, expressed on epithelial cells, common target of infection [9–11]. Interleukin 1 alpha (IL-1α) diverse pro-inflammatory cytokine that share several biological functions including in the encouragement of inflammatory response, maintain cellular immunity and provide host defense against infection [9, 12]. Tumor necrosis factor alpha (TNF-α) occupied in pathological progression of malignant diseases and chronic inflammation, commonly perceived in biopsies of human cancer, which is formed either by epithelial tumor cells such as renal cancer, breast cancer and particularly in this study, ovarian cancer.

The aim of the present study is to investigate the key processes involved in matrix metalloproteinases and inflammatory cytokines in the development of ovarian cancer.

## Materials and Methods

### Sample Collection

The study was conducted at the Institute of Molecular Biology and Biotechnology (IMBB), the University of Lahore and all the selected patients were screened at Inmol Hospital, Lahore. Patients clinically diagnosed with ovarian cancer were included in this study. The subjects with the history of taking drugs (including alcohol and cigarette) and medications (e.g. antiparkinsonian/antipsychotic), were excluded from this study. None of the controls were on any medication, history of chronic infections, malnutrition syndrome, depression, psychosis or metabolic dysfunction (such as diabetes mellitus, liver diseases, cancer) that could interfere with their oxidative metabolites. Fifty female patients of age ranges from 20 to 50 years were recruited in this study. Informed written consent was obtained from patients and healthy participants before being included in this study. Twenty clinically apparently healthy females were included as controls.The experimental protocols were approved by the Research Ethical Committee of the University of Lahore. Five ml of venous blood sample was taken in gel tube from the antecubital vein of each participant at morning time 10.00–11.00 am. The sample tubes were centrifuged at 4000 rpm within two hour of blood collection, after which the serum was separated and stored at -70°C until assayed.

### Analytical Analysis

Glutathione (GSH) assay was performed by the methods of Moron et al., [13]. Superoxide dismutase (SOD) and catalase activities were measured by Kakkar et al., and Aebi and Bergmeyer, respectively [14, 15]. Malondialdehyde (MDA) and nitric oxide (NO) were measured by the methods of Ohkawa *et al*., and Moshage *et al*., [16, 17]. Glutathione peroxidase (GPX) analyzed by Goldberg and Spooner [18]. Vit-E and Vit-A measured by Rosenberg and Culik, and Rutkowski and Grzegorczyk respectively [19, 20]. AOPPs and AGEs (Advance glycation end products) performed by Skrha et al., [21]. TNF-α and IL-1α were measured by commercially available kits (Diaclone Human TNF-α and IL-1 IL-1α ELIZA Kit). MMP-9, MMP-2 and MMP-11 analyzed by commercially available kits (Glory Science Human MMP-9, MMP-2, MMP-11, ELIZA Kits).

### Statistical Analysis

SPSS version 20 was used for the statistical analysis. Results were articulated by mean and standard deviations. The correlation matrix between various biomarkers was also determined. *P* < 0.05 was considered as statistical significance. The results of all parameters were analyzed by independent sample t-test.

## Results

### Circulating Stress Biochemical Markers Profile

**Fig 1:**
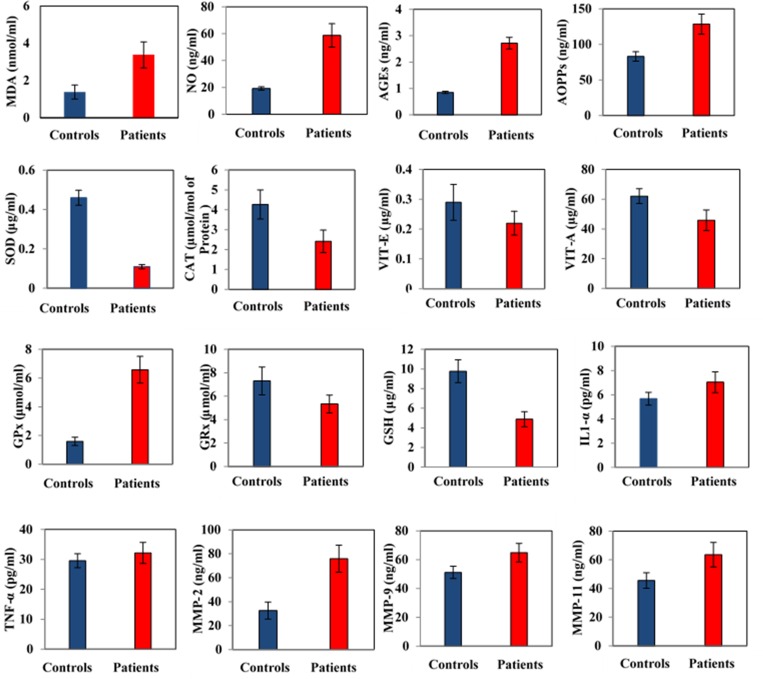
Prognostic variables of ovarian cancer.

The Stress biomarkers profile of ovarian cancer patients e.g., MDA (Malondialdehyde), NO (Nitric oxide), AGEs (Advance glycation end products) and AOPPs (Advance oxidation protein products) shows highly significant difference between control and ovarian cancer patients (Fig 1). Lipid peroxidation is assessed by TBARS and the mean value of MDA in ovarian cancer subjects was elevated at 3.38±1.12 nmol/ml versus (vs) control group at 1.38±0.38 nmol/ml. The mean value of NO in the ovarian cancer subjects is remarkably increased (58.71±8.67 ng/ml) vs control group (19.19±1.31 ng/ml). The serum AGEs value of ovarian cancer patients was also noted to be increased (2.72±0.22ng/ml) as compared to control group (0.85±0.04ng/ml). The mean values of AOPPs indicates higher serum level in ovarian cancer subjects (128.48±27.23 ng/ml), in comparison to control group (83.05±6.63 ng/ml)[Table 1].

**Table 1:** Oxidative Stress, Inflammatory Biomarkers and Matrix Metalloproteinases Status in Ovarian Cancer Patients.

S. No	Parameters	Controls	Patients	*P* Value
1.	MDA (nmol/ml)	1.38±0.38	3.38±1.12	0.005*
2.	NO (ng/ml)	19.19±1.31	58.71±8.67	0.025*
3.	AGEs (ng/ml)	0.85±0.04	2.72±0.22	0.023*
4.	AOPPs (ng/ml)	83.05±6.63	128.48±27.23	0.011*
5.	SOD (μg/ml)	0.46±0.16	0.11±0.08	0.015*
6.	Catalase (μmol/mol of protein)	4.27±0.73	2.41±1.01	0.022*
7.	Vitamin E (μg/ml)	0.29±0.06	0.22±0.04	0.009*
8.	Vitamin A (μg/ml)	62.08±4.91	45.85±9.07	0.003*
9.	GPx (mmol/dl)	1.59±0.29	6.58±0.20	0.004*
10.	GRx (μmol/ml)	7.30±1.19	5.33±1.26	0.017*
11.	GSH (μg/ml)	9.77±1.17	4.88±1.18	0.039*
12.	IL-1α (pg/ml)	5.68±0.53	7.04±0.85	0.000*
13.	TNF-α (pg/ml)	29.57±1.22	32.17±3.52	0.011*
14.	MMP-2 (ng/ml)	32.58±7.18	75.87±18.82	0.029*
15.	MMP-9 (ng/ml)	51.13±7.78	64.87±12.82	0.018*
16.	MMP-11 (ng/ml)	45.60±9.67	63.58±8.48	0.001*

* Significant (p-value <0.05)

### Antioxidant Biomarkers Profile

The Table 1 shows the antioxidants biomarkers profile of ovarian cancer subjects. The mean serum level of SOD (Superoxide dismutase) decreases in ovarian cancer subjects (0.11±0.08μg/ml) vs in controls (0.46±0.16 μg/ml). The lower level of Catalase in ovarian cancer patients was recorded (2.41±1.01 μmol/mol) vs controls (4.27±0.73 μmol/mol). The Vitamin E in ovarian cancer patients was found lower (0.22±0.04 ug/ml) while in healthy controls (0.29±0.06 ug/ml). The low levels of Vitamin A was found (45.84±9.07 μg/ml) in ovarian cancer patients while in control subjects was (62.08±4.91 μg/ml). Higher levels of GPX (Glutathione peroxidase) observed in patients (6.58±0.21μmol/ml) vs healthy controls (1.59±0.29μmol/ml). Decreased GRx level (Glutathione reductase) measured in ovarian cancer patients' (5.33±1.26 mmol/ml) as compare to healthy person's (7.30±1.19 mmol/ml). Similarly, the level of Glutathione (GSH) in patient group was decreased (4.88±1.18 μg/ml) vs control group (9.77±1.17 μg/ml).

### Inflammatory Biomarkers Profile

The data represented in Table 1 summarized the Inflammatory biomarkers profile of IL-1α (interleukin-1α) and TNF-α (tumor necrosis factor-α) in ovarian cancer patients compared with control subjects. The mean value of IL-1α was observed higher (7.04±0.85 pg/ml) in ovarian cancer patients in comparison to control subjects (5.68±0.53 pg/ml). In TNF-α, mean value among ovarian cancer patients was increased (32.17±3.52 pg/ml) vs healthy controls (29.57±1.22 pg/ml).

### MMPs Biomarkers Profile

Table 1 shows the MMPs (Matrix metalloproteinases) profile of ovarian cancer patients and controlled subjects. The mean value of MMP-2 in ovarian cancer patients increased remarkably (75.87±18.82 ng/ml) as compare to healthy controls (32.58±7.18 ng/ml). In ovarian cancer patients, MMP-9 serum levels were observed higher (64.87±12.82 ng/ml) vs control individuals' (51.13±7.78 ng/ml). MMP-11 in ovarian cancer patients were found elevated (63.58±8.48 ng/ml), vs control subjects (45.60±9.67 ng/ml).

## Discussion

This study was performed to evaluate the role of different matrix metalloproteinases (MMPs), oxidative stress parameters, profile of antioxidants and inflammatory markers in the ovarian cancer. MMPs, zinc dependent endopeptidases, are produced according to the cell's requirement by wound cells as well as inflammatory cells and play an important role in both normal and disease conditions. Its activation can be induced by inflammatory growth factors and cytokines controlling the gynecological cancer development by involving in the degradation of the matrix including its components, vitronectin, fibronectin and collagen type I, which contribute to cancer cell adhesion and invasion [8, 22]. It has been revealed that expression of matrix metalloproteinase 2 in ovarian cancer patients with advance tumor and metastasis was raised as compared to controls [23, 24]. The research carried out by Noel et al., reported that MMP-11 expression is elevated in cancer patients and has little impact on tumor progression [25]. This work is similar to the current study in which MMP-11 level is high in ovarian cancer patients as compare to healthy controls. In the present study, MMP-11 shows a statistically strong positive significant correlation with GPX (r = 0.384**) and inverse correlation with SOD (r = - 0.423**)[Table 2].

**Table 2:** Pearson's Correlation Coefficients of Different Variables in Ovarian Cancer Patients.

	SOD	GSH	CAT	VIT.E	NO	IL1α	TNFα	MMP11	MMP9	MMP2	GRx	GPx	AOPPs	AGEs	VIT.A
**MDA**	0.002 0.989	0.079 0.588	0.005 (0.973)	0.134 0.410	0.215 0.133	-0.002 0.987	-0.095 0.559	0.177 0.220	0.255 0.074	0.207 0.149	0.082 0.573	0.166 0.251	**0.273 0.056**	0.085 0.557	0.055 0.703
**SOD**		0-.029 0.840	-0.243 0.089	0.102 0.531	-0.314* **0.026**	0.012 0.935	-0.080 0.624	- 0.423** **0.002**	-0.070 0.631	-0.140 0.331	0.201 0.162	-0.249 0.081	0.168 0.243	0.083 0.566	-0.155 0.282
**GSH**			-0.028 0.849	0.125 0.442	-0.320* **0.023**	0.062 0.668	0.192 0.236	-0.063 0.665	0.103 0.478	0.178 0.215	-0.125 0.388	-0.118 0.415	-0.005 0.971	0.139 0.336	-0.037 0.800
**CAT**				-0.14 0.385	0.242 0.090	-0.110 0.445	0.362* **0.022**	-0.023 0.877	0.178 0.217	0.096 0.509	-0.047 0.746	0.058 0.690	-0.083 0.568	-0.091 0.530	0.327* **0.021**
**VIT.E**					-0.078 0.632	0.094 0.564	-0.471** **0.002**	0.074 0.650	0.073 0.655	-0.199 0.219	0.034 0.834	-0.207 0.201	0.116 0.476	-0.086 0.597	0.100 0.538
**NO**						0.018 0.901	0.047 0.772	0.011 0.939	0.037 0.798	0.066 0.649	-0.100 0.490	0.289* **0.042**	0.154 0.284	0.024 0.866	0.412** **0.003**
**IL1α**							-0.190 0.239	0.000 0.996	0.093 0.520	0.044 0.763	0.123 0.394	0.104 0.474	0.200 0.164	0.114 0.431	-0.283* **0.047**
**TNFα**								-0.200 0.216	-0.111 0.497	0.325* **0.041**	0.123 0.448	-0.116 0.477	-0.097 0.552	-0.097 0.550	0.176 0.277
**MMP11**									0.109 0.451	0.156 0.281	-0.072 0.618	0.384** **0.006**	0.152 0.292	-0.143 0.321	0.105 0.470
**MMP9**										0.218 0.129	0.083 0.567	0.232 0.105	0.132 0.361	0.017 0.904	-0.061 0.675
**MMP2**											0.268 0.060	0.486** **0.000**	0.025 0.862	-0.039 0.789	-0.298* **0.035**
**GR**												-0.006 0.970	-0.166 0.250	-0.206 0.151	-0.018 0.901
**GPX**													0.015 0.916	-0.085 0.559	-0.265 0.063

* Significant (p-value <0.05)

Tumor associated macrophages (TAM) produce cytokines like TNF –α and IL1-α which are the key regulators of cancer associated inflammation as they exert various pro-tumoral activities like monocyte guidance to tumoral tissues, growth factors for tumor cells and adaptive immune reaction suppression. Tumor necrosis factor alpha (TNF-α) is implicated in the upholding and homeostasis of the immune system, inflammation and host defense. The research carried out by Naylor et al., and Colvin, demonstrated that TNF-α has a major function in ovarian cancer development that resembles present study in which there was a significantly increase in levels of TNF-α in ovarian cancer patients as compare to healthy individuals showing a significant positive correlation with MMP-2 (r = 0.325**)[26, 27]. The research carried out by Pollard, and Zeisler*et al*., reviewed the measurement of interleukin 1-alpha (IL-1α) in the serum of ovarian cancer patients and concluded that there is increase level in the ovarian cancer patients as compared to controls [10, 28].

The end product of lipid peroxidation is malondialdehyde (MDA), generated by decomposition of arachidonic acid and larger poly unsaturated fatty acids (PUFAs) and in this study there is increased level of MDA was observed in ovarian cancer patients as compare to controls. Nayak and Kumaraguruparan also reported elevated MDA levels in their studies previously [29, 30]. Advanced oxidation protein products (AOPPs) are dityrosine containing biomarkers of oxidative damage which generate under stressed state and triggers inflammatory processes [31]. In the present study, significantly increased levels of advanced oxidative protein products (AOPPs) was found in patients as compared to controls that results in the increased production of hypochlorous acid by myeloperoxidase enzyme under oxidative and carbonyl stress condition which react with albumin, the most abundant protein found in human blood and oxidizing it to AOPPs which through RAGE receptor trigger NADPH oxidase. Thus, augment the oxidative stress, and inflammatory process. Advanced glycated end products (AGEs) have been recognized in cancerous tissues which led to the generation of cancer tumor. The present study also established result in which the mean value of AGEs is significantly increases in ovarian cancer patients as compare to healthy individual [32].

Nitric oxide (NO) has dual function; it can endorse and inhibit tumor initiation and metastasis. Increased and unrestricted level of nitric oxide leads toward death by promoting definite illness [33]. The research carried out by scientist stated that NO and nitric oxide synthase (NOS) expression in many tumors is increased as compared to normal tissues and these findings also confirmed in the current study [34]. SOD shows a statistically significant inverse correlation with nitric oxide (r = -0.314*) and MMP-11 (r = -0.423**) in present study, also researched and confirmed in some previous studies [35]. Glutathione (GSH) is decreased in ovarian cancer patients as compare to healthy individuals and this work is similar according to the study of Maher [36]. GSH show a statistically significant negative association with NO (r = -0.320*). The past researches including Skrzydiewska et al., reported that in ovarian cancer patients antioxidant enzyme GPx activities are significantly increased as compare to control, that conform with our results [37, 38]. GPX also showed a statistically significant positive association with NO (r = 0.289*), MMP2 (r = 0.486**) and MMP 11(r = 0.384**).

Vitamin A is a nutritional unsaturated organic compound which consists of retinal, retinoic acid, retinol, provitamin A, carotenoids and beta-carotene [39]. In the present study, vitamin A observed to be significantly decreased in ovarian cancer patients as compared to normal individuals. Tissue damage and disease aggravation leads to the lower levels of Vitamin A [40–43]. Vitamin A has positive correlation with catalase (r = 0.327*) and NO (r = 0.412**). Vitamin E also called as alpha-tocopherol is lipid soluble, non-toxic and chain breaking antioxidants in cellular membrane which provide the protection to the cell membrane from reactive oxygen species generated by oxidative stress [44]. Present study reports the Vitamin E levels are decreased in the ovarian cancer patients as compare to healthy group, and this fact is also established in previous studies, and resultantly this decline of vitamin E leads to the cell membrane damage [45]. The resultant oxidative insult induces MMP gene expression by triggering RAS oncogenes, MAPK family and P38, thus hampering phosphatase activity.

## Conclusions

Several lines of evidence indicate that MMPs are frequently expressed in the ovarian cancer having a key role in metastasis and also are potential prognostic markers. Cytokines released by tumor associated macrophages (TAM) and oxidative insult found in tumors are more likely to contribute in cancer progression as both have been implicated in MMPs gene expression. Thus, targeting not only cytokines and oxidative insult but also MMPs by tissue inhibitors of metalloproteinases (TIMPs) are the valuable therapeutic approaches to complement conventional anticancer strategies.
